# Visualization of three-dimensional microcirculation of rodents’ retina and choroid for studies of critical illness using optical coherence tomography angiography

**DOI:** 10.1038/s41598-021-93631-9

**Published:** 2021-07-12

**Authors:** Jang Ryul Park, ByungKun Lee, Min Ji Lee, Kyuseok Kim, Wang-Yuhl Oh

**Affiliations:** 1 Department of Mechanical Engineering, Korea Advanced Institute of Science and Technology (KAIST), 291 Daehak-ro, Yuseong-gu, Daejeon, 34141 Republic of Korea; 2 KI for Health Science and Technology, Korea Advanced Institute of Science and Technology (KAIST), 291 Daehak-ro, Yuseong-gu, Daejeon, 34141 Republic of Korea; 3 Department of Emergency Medicine, CHA University School of Medicine, Seongnam, 13497 Republic of Korea

**Keywords:** Optical imaging, Biomedical engineering, Imaging and sensing

## Abstract

We developed a method to measure the relative blood flow speed using optical coherence tomography angiography (OCTA) in retina and choroid, and investigated the feasibility of this method for assessing microcirculatory function in rat models of sepsis and hemorrhagic shock. Two sepsis models, 6-h severe sepsis without treatment and 30-h moderate sepsis maintaining mean arterial pressure, and volume controlled hemorrhagic shock and fluid resuscitation model were used to see the change of microcirculation. The blood flow index (BFI), which was calculated from the OCTA images to represent the average relative blood flow, was decreasing during the 6-h severe sepsis model. Its change is in parallel with the mean arterial blood pressure (MAP) and blood lactate levels. In the 30-h moderate sepsis model, the BFI was decreased while maintaining MAP, and lactate was increased. In the hemorrhagic shock model, the change of BFI is in line with MAP and lactate levels. In all models, BFI change is more sensitive in choroid than in retina. This study presents the OCTA-based retinal and choroidal microcirculatory blood flow monitoring method and shows its utility for assessment of critical illness.

## Introduction

Sepsis and hemorrhagic shock are significant life-threatening public health concerns, leading 30% or more of the patient population to death^1–3^. Since sepsis or hemorrhagic shock can rapidly progress into fatal conditions, early and accurate diagnosis followed by a timely treatment is crucial for patient survival. Blood flow in the microvasculature, namely the network of arterioles, venules, and capillaries under 100-μm vessel diameter, is directly related to cellular-scale metabolic transport necessary for proper cell function. Multiple studies suggested that alterations of the microcirculation play a crucial role in critical illness^4–6^. However, the microcirculatory function cannot be directly monitored by conventional clinical measurements such as blood pressure, cardiac output, arterial blood lactate, and urine examination^7,8^ because these indicators represent the gross-scale circulatory function. Therefore, the assessment of microcirculatory function can facilitate early diagnosis and improved prognosis of critical illness by providing critical information about organ and tissue perfusion and metabolic transport.

Optical coherence tomography (OCT) can perform micron-scale resolution, non-contact, three-dimensional imaging of the retina and the choroid in vivo. Advances in wavelength-swept laser and line-scan camera technologies in the last decade have enabled high-speed OCT imaging above 100 kHz axial scan rate, allowing label-free angiographic imaging using motion contrast generated by detecting the OCT signal variation. OCT angiography (OCTA) uses scan patterns with rapidly repeated B-scans to estimate statistical variance of the OCT signal magnitude or phase to extract information on the presence or absence of blood flow and the relative blood flow speed^9–14^.

In this study, we investigated the utility of quantitative angiographic imaging in the retina and the choroid using OCT for assessing microcirculatory function in rat models of sepsis and hemorrhagic shock. The rats were monitored longitudinally with periodic OCTA imaging and the relative blood flow change was quantified using a blood flow index (BFI) defined based on OCTA imaging.

## Results

### Multi-interscan-time OCT angiography and BFI analysis

**Figure 1:**
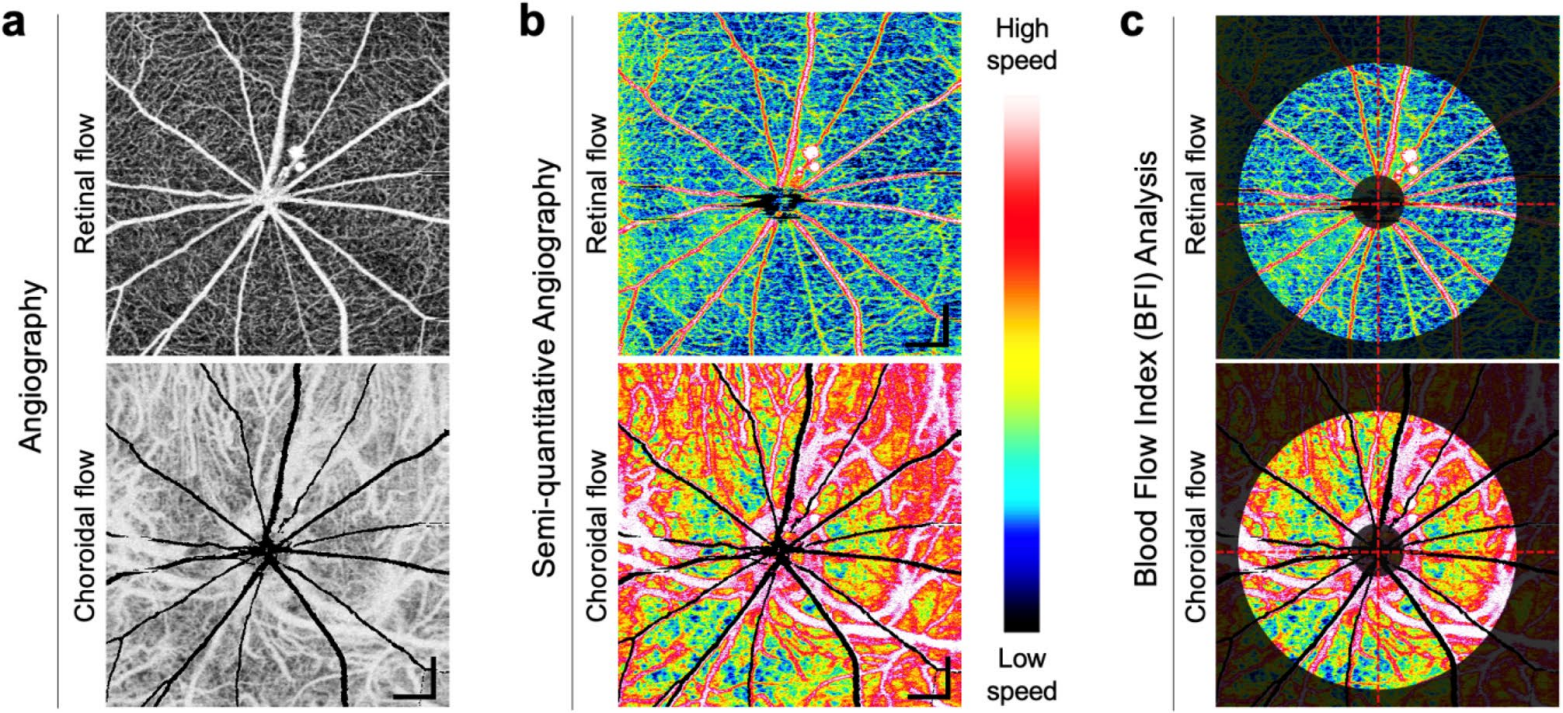
Multi-Interscan-Time OCT Angiography and Blood flow index (BFI) analysis. (**a**) Shows conventional *en face* OCT angiograms of the retinal and choroidal layers visualizing vascular morphology. (**b**) Shows semi-quantitative OCT angiograms providing color-coded display of relative flow speeds in the capillaries of the retinal and the choroidal layers using multi-interscan-time OCTA. Retinal and choroidal *en face* angiograms were generated by segmenting the line corresponding to the RPE-Bruch membrane complex and projecting, respectively. Shadow artifacts caused by thick retinal vessel in the choroidal layer are shaded in black. The BFI’s for the retinal and the choroidal flows were calculated in the donut-shaped area covering 0.5 mm through 2.5 mm distance from center of the optic nerve head (**c**). Scale bars: 400 μm.

We have acquired multi-interscan-time OCTA images of the posterior eye in normal rats to validate our imaging and visualization methods. Retinal and choroidal *en face* angiograms were generated by segmenting the line corresponding to the RPE-Bruch membrane complex and projecting the pixels above and below the line, respectively. Figure 1a shows conventional *en face* OCT angiograms of the retinal and the choroidal layers. Figure 1b shows that color-coded display of relative flow speeds using multi-interscan-time OCTA can facilitate intuitive judgment of overall capillary blood flow in the retina and the choroid. The BFI’s for the retinal and choroidal flows were calculated separately in the donut-shaped ROI covering 0.5 mm through 2.5 mm distance from the center of the optic nerve head as shown in Fig. 1c.

### Blood flow in 6-h severe rat sepsis model

**Figure 2:**
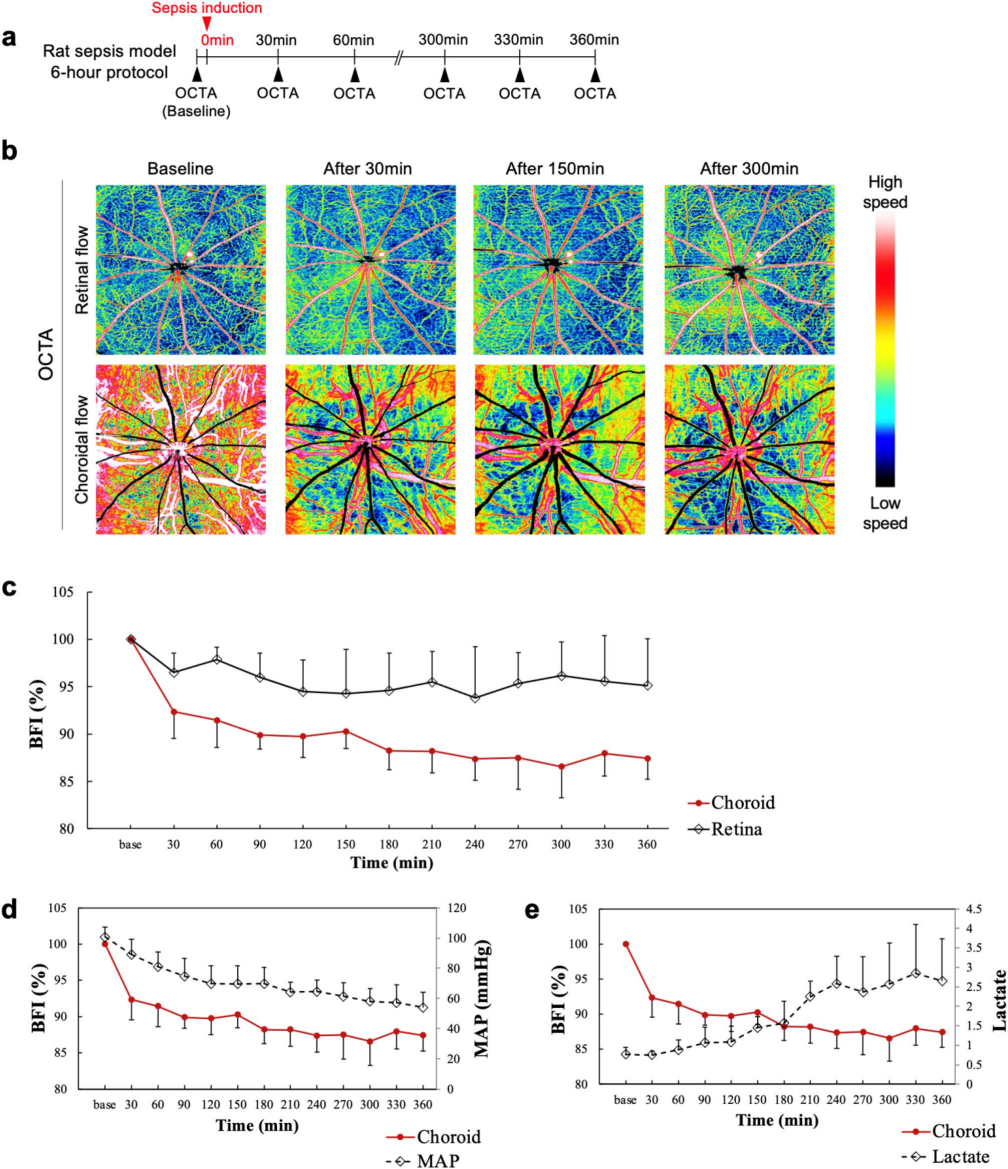
Blood flow change in 6-h severe rat sepsis model. For measuring acute change in blood flow speed, the animal received 7 ml/kg fecal slurry injection and underwent OCTA imaging every 30 min for 6 h (**a**). (**b**) Shows color-coded multi-interscan-time OCTA images of the retinal and choroidal layers from the 6-h protocol. The retinal and choroidal BFI value (mean ± SD, n = 5) changes over time are shown in (**c**). (**d**,**e**) Show the choroidal BFI change in parallel with mean arterial blood pressure (MAP) and blood lactate, respectively. Changes in BFI, MAP, and lactate are summarized in Table 1.

Figure 2b shows color-coded multi-interscan-time OCTA images from the 6-h protocol of severe sepsis model (Fig. 2a). Figure 2c shows time versus BFI value (mean ± SD, n = 5) of retinal and choroidal flow. Retinal BFI at 360 min after sepsis induction was 95.1% of the baseline value; on the other hand, choroidal BFI dropped to 92.3% at 30-min mark and 87.4% at 360-min mark. While retinal flow did not change significantly during the course of septic shock, choroidal flow already showed a noticeable decrease at the 30-min mark and dropped further with time. Retinal and choroidal BFI plots in the individual animals are shown in Fig. 2. Figure 2d,e show BFI change in parallel with other vital records such as mean arterial blood pressure (MAP) and blood lactate. MAP gradually decreased from 100.7 to 54 mmHg and blood lactate gradually increased from 0.77 to 2.65 over 360 min. Changes in BFI, MAP, and lactate are summarized in Table 1.

**Table 1:** Changes of BFI, MAP, and lactate in 6-h severe rat sepsis model.

Parameter (unit)	Baseline	30 min	90 min	150 min	210 min	270 min	330 min
BFI-retina (%)	100 (0)	96.5 (2)	96 (2.6)	94.3 (4.6)	95.5 (3.2)	95.3 (3.3)	95.6 (4.8)
BFI-choroid (%)	100 (0)	92.3 (2.8)	89.9 (1.5)	90.3 (1.8)	88.2 (2.3)	87.5 (3.3)	88 (2.4)
MAP (mmHg)	100.7 (6.7)	89.2 (10.1)	74.7 (11.9)	69.7 (11.9)	64.2 (6.7)	61.3 (9.2)	57.2 (11.7)
Lactate (mmol l^−1^)	0.77 (0.18)	0.75 (0.1)	1.07 (0.39)	1.45 (0.29)	2.25 (0.4)	2.37 (0.91)	2.85 (1.25)

### Blood flow in 30-h moderate rat sepsis model

Figure S4b shows the plot showing time versus BFI value (mean ± SD, n = 5) from the 30-h protocol of moderate sepsis model (Fig. S4a). At the 24-h mark, the mean BFI was 98.8% in the retina and 95.7% in the choroid. While the retinal BFI was sustained at 98.0% at the 29.5-h mark, the choroidal BFI started to gradually decrease from the 26-h mark and reached to 90.9% at the 29.5-h mark. Figure S4c,d show choroidal BFI overlaid with MAP and blood lactate measurements at each time point, respectively. The measurements demonstrate that the MAP was stable at around 74.15 mmHg during the final 6 h of the protocol. The dose rates of norepinephrine for each animal are summarized in supplementary table 1. In all 5 animals, choroidal BFI appeared to be recovered at the usage of the pressor agent but quickly returned to the gradually decreasing trend. BFI, MAP, and blood lactate changes in the individual animals are shown in Fig. S5.

### Blood flow in rat hemorrhagic shock model

**Figure 3:**
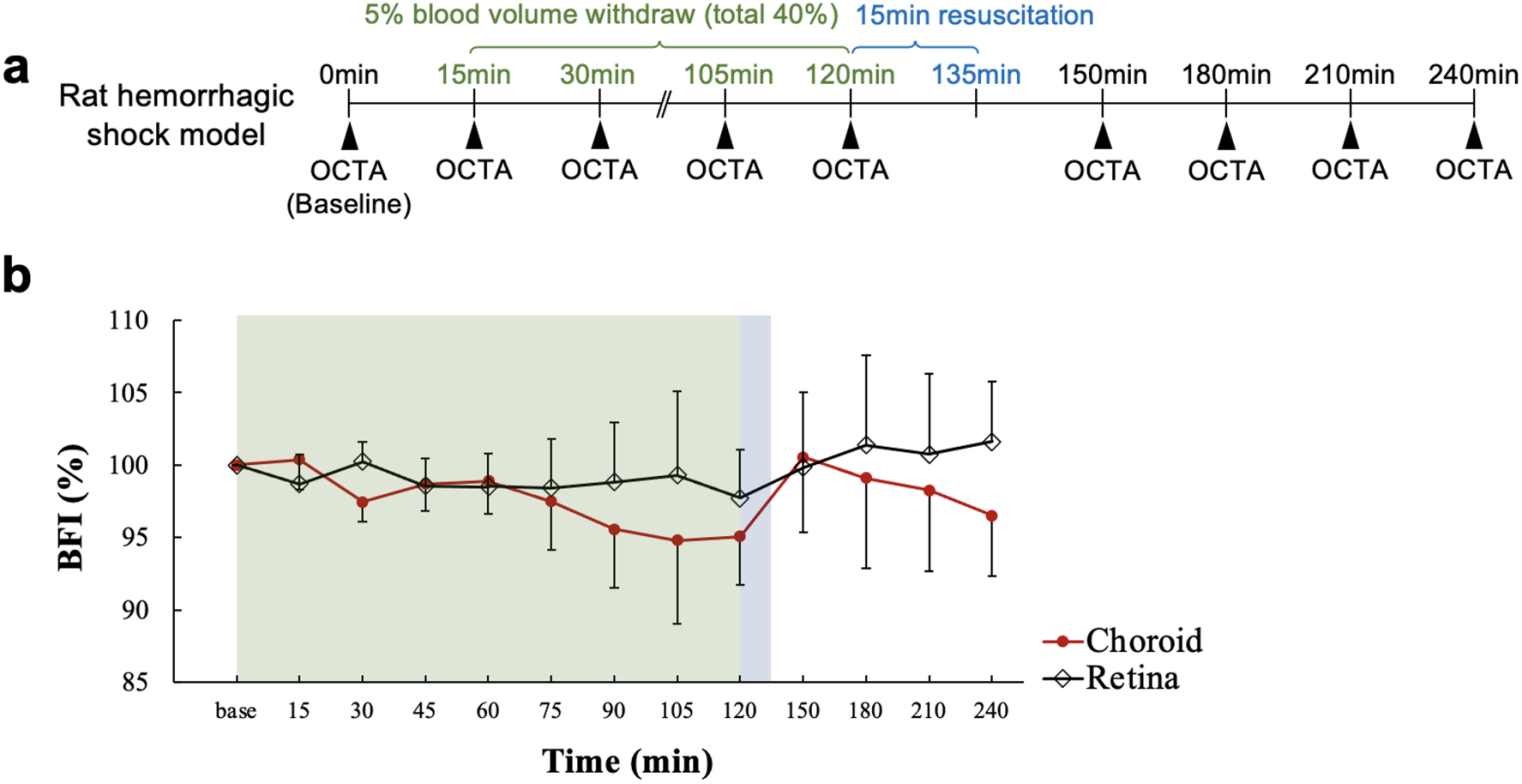
Blood flow change in rat hemorrhagic shock model. In order to simulate hemorrhagic shock in the rat, 5% of blood volume was withdrawn eight times with a 15-min interval in the first two hours and then resuscitation was performed for 15 min. OCTA imaging was performed right after every blood withdrawal and every 30 min for 1.5 h after resuscitation (**a**). (**b**) Shows time versus BFI values (mean ± SD, n = 5) of the retinal and the choroidal flows. Changes in BFI, MAP, and lactate are summarized in Table 2.

Figure 3b shows time versus BFI value (mean ± SD, n = 5) from the 40% blood loss hemorrhagic shock model (Fig. 3a). After the 2-h long blood withdrawal period, the retinal BFI was 97.7%, whereas the choroidal BFI was decreased to 95.1%. MAP was decreased to 64 mmHg. After resuscitation, the retinal BFI gradually increased to 101.6%, while the choroidal BFI first increased to 100.5% and then decreased back to 96.5%. After resuscitation, the MAP increased up to 90.2 mmHg and decreased back to 72.4 mmHg (Fig. S3b). Blood lactate gradually increased from 0.8 to 1.78 throughout the process (Fig. S3c). These results are summarized in Table 2.

**Table 2:** Changes of BFI, MAP, and lactate in hemorrhagic shock model.

Parameter (unit)	Baseline	15 min	60 min	105 min	120 min	180 min	240 min
BFI-retina (%)	100 (0)	98.7 (0.9)	98.5 (1.8)	99.3 (0.8)	97.7 (5.1)	101.4 (2.6)	101.6 (2.4)
BFI-choroid (%)	100 (0)	100.4 (2.1)	98.9 (2.3)	94.8 (5.8)	95.1 (3.4)	99.1 (6.2)	96.5 (4.2)
MAP (mmHg)	101.4 (13.6)	89.4 (1.9)	83.8 (8.5)	65.2 (15.2)	64 (0)	78.8 (9.3)	72 (6.3)
Lactate (mmol l^−1^)	0.8 (0.13)	0.96 (0.16)	1.34 (0.72)	1.74 (1.39)	1.15 (0.15)	1.66 (1.17)	1.78 (1.34)

## Discussion

We have demonstrated that the multi-interscan-time OCTA can detect retinal and choroidal capillary blood flow speed changes in rat sepsis and hemorrhagic shock models. While the measurement of retinal capillary flow speed change using a single-interscan-time OCTA has been previously demonstrated^8^, we have presented measurement of wide range capillary flow speed changes using multi-interscan-time OCTA in the retina and the choroid in rat models of sepsis and hemorrhagic shock. This is well aligned to the pathophysiological understanding that microcirculatory dysfunction would play an important role in sepsis and hemorrhagic shock.

Retinal and choroidal BFI were calculated and longitudinally tracked along with MAP and blood lactate to investigate capillary flow speed change in the presence of sepsis or hemorrhagic shock. In the 6-h severe sepsis model, the choroidal BFI decreased immediately and showed a gradually decreasing trend over time. It seemed more sensitive compared to MAP and lactate level. In the 30-h moderate sepsis model, we tried to maintain MAP 75 mmHg to see the loss of coherence between macro- and microcirculation^15–22^. The choroidal BFI seemed to be temporarily recovered by the usage of the pressor agent but quickly returned to the decreasing trend. This result suggests that the microcirculation can be still compromised even when the arterial pressure is sustained, which implies the importance of monitoring of microcirculation in sepsis. In the hemorrhagic shock model, the choroidal BFI steadily decreased during the withdrawal period and appeared to return to the baseline after the initial reperfusion. However, the BFI still decreased during the continuing reperfusion period.

Interestingly, in all our measurements in the rat disease models, choroidal BFI manifested a more pronounced change than the retinal BFI. Several factors can cause this trend. First, retinal blood flow autoregulation could be more responsive to external change than the choroidal blood flow autoregulation. Since the retina is a part of the central nervous system, retinal capillary blood flow could be autoregulated at a relatively constant metabolic supply rate^23–25^. In contrast, choroidal blood flow is known to be richly innervated by the autonomous nerve fibers and trigeminal sensory nerve fibers and less influenced by autoregulation^26^. This difference may be the key reason for the clear difference between retinal blood flow and choroidal blood flow changes in sepsis and hemorrhagic shock models. Second, the difference in the absolute flow speed inside the retinal and choroidal vessels can affect the sensitivity of BFI for detecting blood flow changes. Retinal BFI is calculated from retinal vessels with various calibers, while choroidal BFI mostly depends on the densely packed, honeycomb-shaped choriocapillaris layer. Therefore, choroidal BFI better represents slow capillary flow more heavily than the retinal BFI. Taken together, the microcirculatory dysfunction could be detected more sensitively in the choroidal flow rather than in the retinal flow.

Quantitative measurement of microcirculatory function can be performed at multiple locations. Three of the most obvious and easiest targets had been the microcirculation in the skin, skeletal muscle, and sublingual glands^7,8,27,28^. Currently, the most widely accepted imaging method for measurement of microcirculatory function is hand-held vital microscopes (HVMs) including sidestream dark field (SDF) and incident dark field (IDF) imaging. HVMs can visualize the motion pattern of individual erythrocytes flowing in the capillaries of the sublingual gland. The efficacy of HVMs-based assessment for the microcirculatory function was demonstrated in various diseases including sepsis^29^. HVMs videos were taken at high time resolution and converted into microcirculatory flow index (MFI), a semi-quantitative measure relying on the reader interpretation. However, there are known limitations of HVMs. The major shortcoming is pressure-induced microcirculatory artifacts which the inventors of SDF imaging mentioned^30^, as SDF imaging requires direct contact between the microscope probe and the tissue. External pressure applied onto the sublingual mucosa can disturb the normal flow in the capillaries enough to appear as turbulent or non-continuous flow. This artifact can result in a considerable false-positive rate of microvascular dysfunction. The measurement of microcirculation in the non-compressible area could be the solution to this limitation, and having said that, the retinal and choroidal microcirculation could be the perfect candidate.

There are three noteworthy limitations of this study. The first one is the absence of an alternative capillary blood flow measurement as a reference standard. However, there are an increasing number of publications supporting validity of OCT-based blood flow measurements. Multiple groups have reported that multi-interscan-time OCTA is capable of measuring relative flow speeds in the same scan and blood flow speed changes in the same vessels^31–35^. In particular, our method of blood flow speed measurement using hybrid scan mCD has been validated in flow phantoms, where the velocity measurements were highly repeatable and accurate^33^. Our sepsis model involves rapid changes in blood flow, which require relatively short imaging time. Moreover, wide range of measurable flow speed is necessary for simultaneous measurement of retinal and choroidal blood flow. Therefore, the authors have concluded that the multi-interscan-time mCD method is a reasonable approach for measuring transient blood flow changes in our sepsis rat model.

The second limitation is the relatively large standard deviation of the BFI results among the individual animals. The animals appeared to have different degrees of pathologic reaction, although all animals were treated with the same protocol. This variation in clinical parameters was also observed in previous studies^36–39^ using the same sepsis induction protocol. A follow-up investigation with larger number of animals could improve the precision of the sample mean of the BFI. Moreover, the precision of BFI as a capillary flow measure should be also validated. OCTA is susceptible to low OCT signal strength, motion artifact, and angular dependence of the non-isotropic resolution. Other factors such as the ROI size and BFI calculation formula can also change the result. The repeatability and reproducibility of BFI should be validated in a separate study before making definitive statements about the capillary flow in the retina and the choroid.

Multi-interscan-time OCTA can be improved with technical development. The mCD for each interscan time is calculated from three repeats. Considering the stochastic nature of blood flow in small vessels and capillaries, a larger number of repeats would make the statistical measurement more precise. This can be potentially achieved with faster imaging systems and faster scanning solutions, whose preliminary results are already published by our group^40^. In addition, there are less well understood secondary factors which can affect the BFI. Factors such as RBC density and blood vessel thickness can potentially affect the BFI significantly. This uncertainty complicates an objective cross-sectional comparison of absolute BFI measurements across different subjects. A multivariate analysis using a more advanced flow phantom such as intralipid could improve our understanding of BFI.

In conclusion, we have used multi-interscan-time OCTA imaging with a prototype SS-OCT system to investigate retinal and choroidal capillary blood flow in rat sepsis and hemorrhagic shock models. OCTA-based BFI could potentially become a measure of microcirculation function. The choroidal BFI appeared to be a better sensor for microvascular dysfunction in critical disease than the retinal BFI.

## Methods

### OCTA system

We used a prototype high-speed OCTA system based on a wavelength-swept laser operating at a 230-kHz repetition rate (Fig. S1a). The laser sweeps over a 94-nm wavelength range centered at 1048 nm, which corresponds to 6.9-µm axial resolution in tissue after digital spectral shaping. With 1.6 mW optical power incident onto the rodent cornea, the imaging sensitivity was 98 dB near the top of the imaging window.

### Scan protocol

OCTA volume data were acquired using a special raster scan pattern with 768 B-scans covering a 3.2 mm × 3.2 mm area centered at the optic nerve head of the rat eye. In order to obtain information about relative blood flow speed, each B-scan location was repeatedly scanned three times, using five different interscan times—the number of A-lines per B-scan was set at 320, 384, 512, 768, and 1024, which correspond to the interscan times of 1.39, 1.67, 2.22, 3.34, and 4.45 ms, respectively (Fig. S1b). To complete scanning with the five inter-scan times, 30.1 s of imaging time was spent per volume.

### OCTA image processing

After the bulk motion between the repeated frames was compensated using subpixel image registration, the mean complex decorrelation (mCD) value^33^ was computed with the five different inter-scan times to estimate relative blood flow speed.

The multi-interscan-time mCD method presented in this study is similar to the variable interscan time analysis (VISTA) previously proposed by Choi et al.^31,41^. VISTA and mCD methods have two clear differences in their details. First, the scan patterns are different. While VISTA used five B-scan repeats with the same number of A-scans per B-scan, our method used a hybrid scan with five different numbers of B-scans. The hybrid scan is advantageous for performing multi-interscan-time OCTA at lower A-scan rates, because the number of A-scans per B-scan can be chosen either for shorter interscan time or for higher pixel resolution. Denser B-scans with longer interscan time are targeted for small capillaries, whereas sparser B-scans with shorter interscan time are useful for detecting fast flow speeds in large vessels. Second, the image processing algorithms are different. VISTA used two OCTA images generated from magnitude decorrelation images with 1.5 ms and 3.0 ms interscan times, respectively. The ratio between the two OCTA images was used as an indicator for blood flow speed. In contrast, our method uses complex OCT signal to calculate mCD, which showed a nearly linear relationship with flow speed in flow phantoms^33^.

For optimized visualization of the *en face* angiograms, the inner plexiform layer (IPL) and the Bruch membrane (BM) were segmented to generate retinal and choroidal projections, respectively. Vascular projection artifacts caused by decorrelation tail under large vessels were removed by masking out the area occupied by large vessels. The mCD value was converted into relative flow speed in an arbitrary unit specific to the subject and displayed in a color scale directly proportional to the absolute flow speed. All aforementioned processing steps were fully automatic and did not require additional user input.

We have introduced a numerical index representing the blood flow speed change from the baseline, in order to perform quantitative time-analysis of blood flow speed in the retina and the choroid. The blood flow index (BFI) is a number that reflects the average relative blood flow speed in a defined region of interest. If we let $V(x,y,t)$ denote the *en face* mean projection of the mCD at time *t*, then the BFI is defined as below:

$$\text{BFI}(t)=\sum_{(x,y)\in ROI}V\left(x,y,t\right)/\sum_{(x,y)\in ROI}V\left(x,y,0\right)$$

where t = 0 refers to the OCTA image measured at the baseline. Note that in the choroidal projections, the areas shadowed by major retinal vessels were excluded because the “decorrelation tail” artifacts can cause flow overestimation. Details about the decorrelation tail are described in “Discussion”. All image processing steps including the BFI calculation were fully automated using a commercial computation platform (Matlab; Mathworks, Natick, MA, USA).

### Animal preparation

Left eyes of Sprague–Dawley rats with body weights ranging from 340 to 360 g were imaged. The animal was anesthetized using Zoletil (30 mg/kg) and Xylazine (10 mg/kg) cocktail before the induction of sepsis or hemorrhagic shock. The femoral artery was catheterized for blood withdrawal, arterial blood pressure measurement, and blood gas analysis. For optimal imaging performance, tropicamide (1%) was applied onto the rat cornea for pupil dilation right after the induction of anesthesia. During OCT imaging, eye drops were applied periodically to prevent corneal drying. The eyelids were fixed open using surgical tape and the outside ambient light was blocked to prevent any retinal damage caused by overbleaching of the photoreceptors. All experiments were conducted in accordance with the Animal Research: Reporting In Vivo Experiments (ARRIVE) guidelines and the ARVO Statement for the Use of Animals in Ophthalmic and Vision Research, with the approval of the Institutional Animal Care and Use Committee (IACUC) of Seoul National University Bundang Hospital (SNUBH).

### Sepsis model

Sepsis was induced by peritoneal administration of rat fecal slurry by amounts of 5 ml/kg or 7 ml/kg as previously demonstrated by Lee et al.^42^. In brief, polymicrobial sepsis was induced using a fecal slurry peritonitis model. We used two models. One is a 6-h protocol of severe sepsis model using 7 ml/kg of fecal slurry and monitored for 6 h immediately after sepsis induction. Another is a 30-h protocol of moderate sepsis model using 5 ml/kg of fecal slurry. Twenty-four hours after sepsis induction, rats were monitored for 5.5 h (Fig. 2a). We administered continuous infusion of norepinephrine to maintain MAP at 75 mmHg.

### Hemorrhagic shock model

Forty percent blood loss hemorrhagic shock model used in a previous study by Fukudome et al. was used^43^. Forty percent of the total blood volume was gradually removed from the femoral artery over eight repeats in 2 h. The blood volume of the animal was decreased by 5% at a time with a 15-min interval. Resuscitation was performed with a 15-min reinfusion of the entire removed portion of the blood volume. The animals 15 min of stabilization, rats were monitored for 1.5 h (Fig. 3a).

### Imaging protocol and measurement of MAP and lactate

OCTA imaging was performed in the rat sepsis model and the hemorrhagic shock model to record the time course of the capillary flow speed change (Figs. 2a and 3a). For the rat sepsis model, the rats were imaged at baseline and 30-min intervals during the sepsis induction and the recovery. For the hemorrhagic shock model, since OCTA imaging was performed right after every blood withdrawal and a reperfusion event, the rats were imaged at baseline and 15 min intervals during the withdrawal period. After resuscitation, the rat underwent OCTA imaging every 30 min for 1.5 h. Mean arterial pressure and arterial lactate were measured directly after every OCTA imaging session.

### Ethics approval and consent to participate

All experiments were conducted in accordance with the Animal Research: Reporting In Vivo Experiments (ARRIVE) guidelines and the ARVO Statement for the Use of Animals in Ophthalmic and Vision Research, with the approval of the Institutional Animal Care and Use Committee (IACUC) of Seoul National University Bundang Hospital (SNUBH).

## Supplementary Information

Supplementary Information.

## Data Availability

The datasets used and/or analyzed during the current study are available from the corresponding author on reasonable request.
