# Supplementary material for: Visualization of three-dimensional microcirculation of rodents’ retina and choroid for studies of critical illness using optical coherence tomography angiography
Source: Sci Rep. 2021 Jul 12;11:14302. doi: 10.1038/s41598-021-93631-9 (PMC8275781; doi:10.1038/s41598-021-93631-9)
Supplement: Supplementary file 1 — Supplementary Information. [file 41598_2021_93631_MOESM1_ESM.docx]

**Supplementary Figure 1.** A schematic of a prototype high-speed OCTA system (Fig. S1(a)) and the beam scan method (Fig. S1(b)). B1, B2, B3, B4, and B5 show OCTA *en face* images acquired with 320, 384, 512, 768, and 1024 A-lines per B-scan, respectively.

**Supplementary Figure 2.** The 6-hour protocol of severe rat sepsis model (Fig. S2(a)) and the changes of the retinal (Fig. S2(b)) and the choroidal (Fig. S2(c)) blood flows in the individual animals.

**Supplementary Figure 3.** Blood flow, mean arterial blood pressure (MAP) and blood lactate changes in rat hemorrhagic shock model. (Fig. S3(b)) and (Fig. S3(c)) show the choroidal BFI change in parallel with MAP and blood lactate, respectively.

**Supplementary Figure 4.** The 30-hour protocol of moderate rat sepsis model (Fig. S4(a)) and the change of the blood flow (Fig. S4(b-d)). Fig. 34(b) shows time versus BFI values (mean+-SD, n=5) of retinal and choroidal flows. The choroidal BFI overlaid with MAP and blood lactate measurements at each point are shown in Fig. S4(c) and (d), respectively. The dose rate of norepinephrine for each individual animal are summarized in supplementary table 1.

**Supplementary Figure 5.** Choroidal BFI, MAP, and blood lactate change in 30-hour moderate rat sepsis model. Changes in MAP (Fig. S5(a1)-(e1)) and blood lactate (Fig. S5(a2-e2)) in the individual animals are shown.

**Supplementary Table 1.** The dose rate of norepinephrine for each individual animal.

Supplementary Figure 1. A schematic of a prototype high-speed OCTA system (Fig. S1(a)) and the beam scan method (Fig. S1(b)).


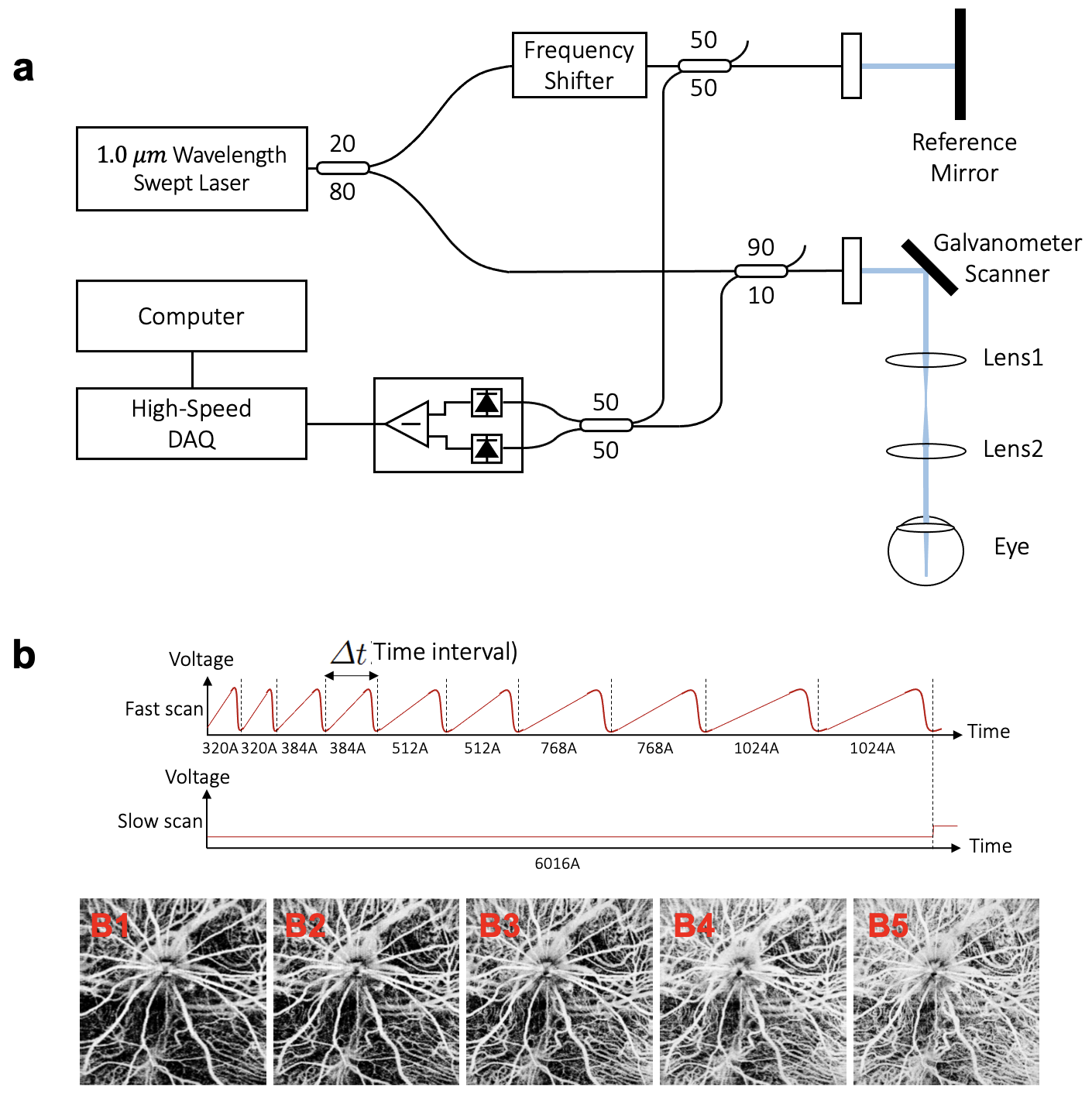


Supplementary Figure 2. The 6-hour protocol of severe rat sepsis model (Fig. S2(a)) and the changes of the retinal (Fig. S2(b)) and the choroidal (Fig. S2(c)) blood flows in the individual animals.


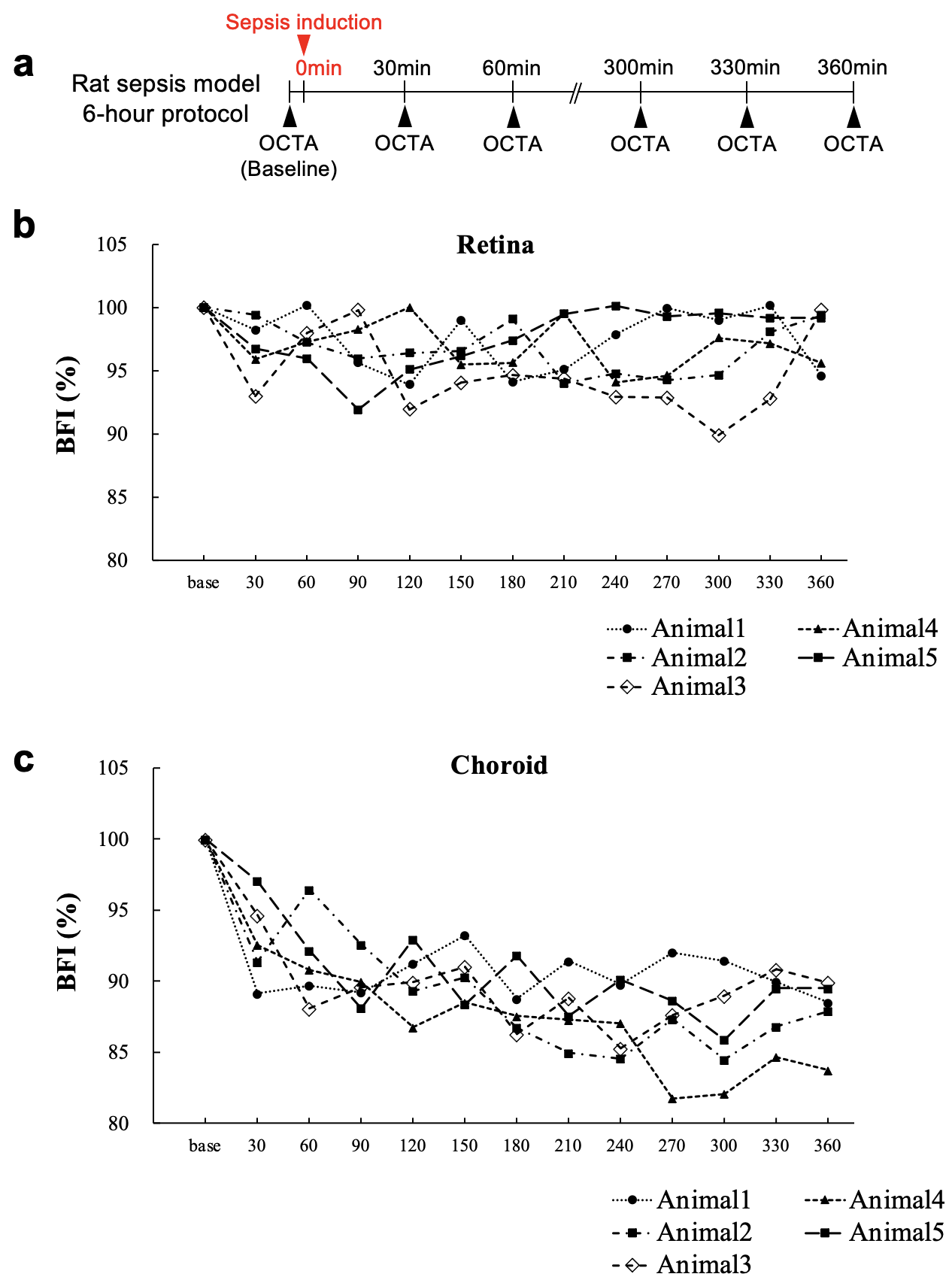


Supplementary Figure 3. Blood flow, mean arterial blood pressure (MAP) and blood lactate changes in rat hemorrhagic shock model.


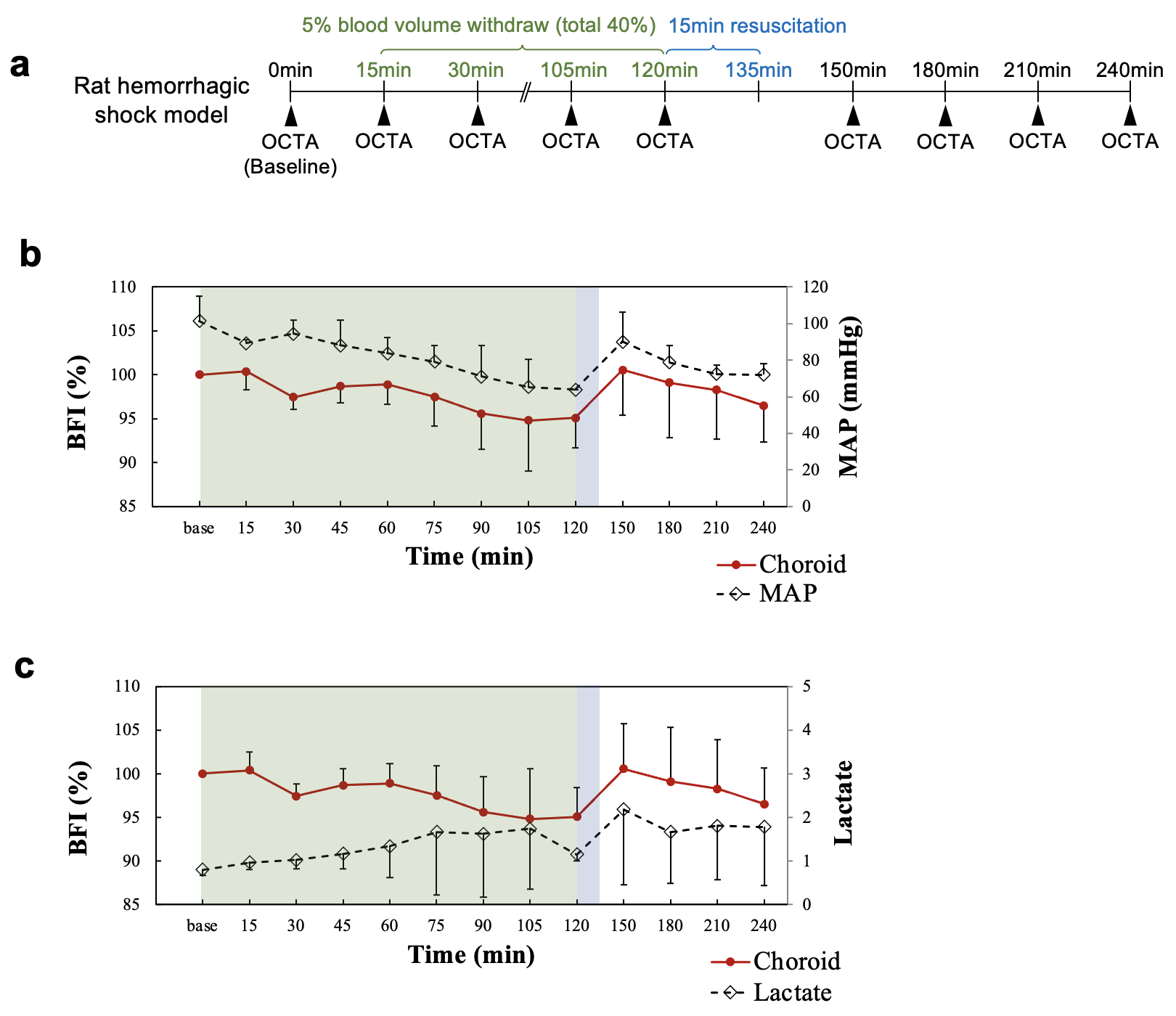


Supplementary Figure 4. The 30-hour protocol of moderate rat sepsis model (Fig. S4(a)) and the change of the blood flow (Fig. S4(b-d)).


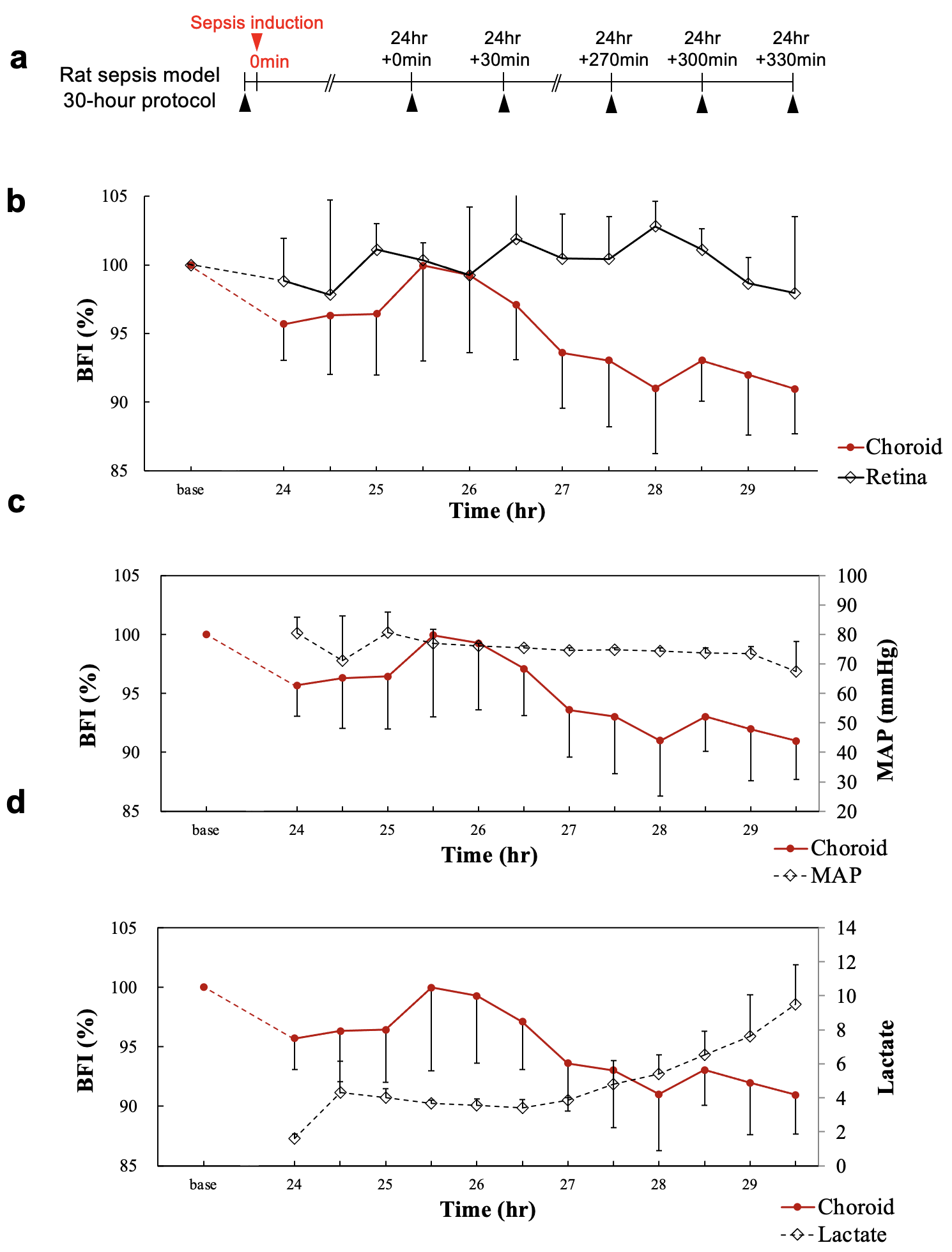


Supplementary Figure 5. Choroidal BFI, MAP, and blood lactate change in 30-hour moderate rat sepsis model.


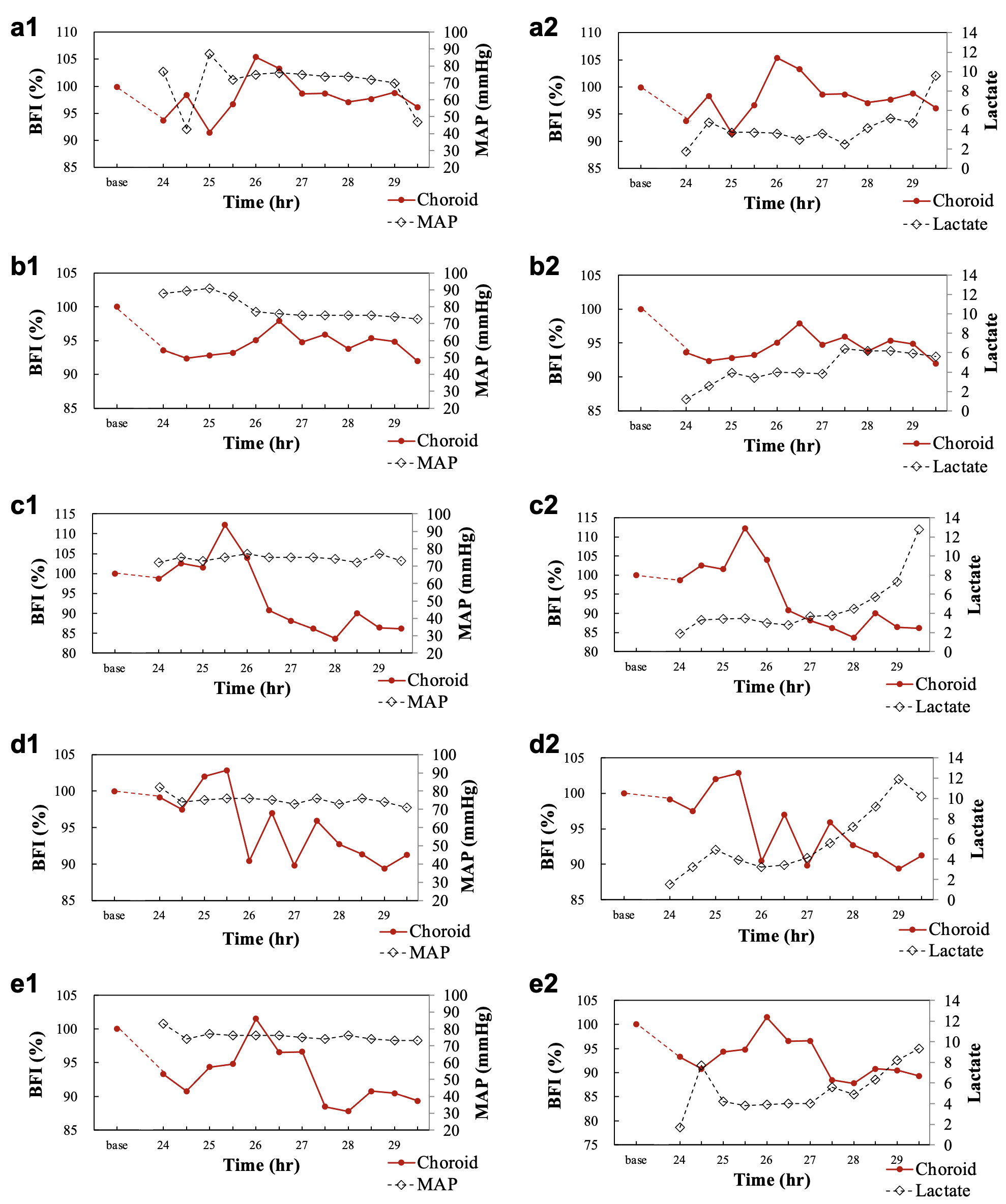


Supplementary Table 1. The dose rate of norepinephrine for each individual animal.

**Dose rate of NE [cc/hr]**

| **Animal** | **Baseline** | **24 hr** | **24.5 hr** | **25 hr** | **25.5 hr** | **26 hr** |
| --- | --- | --- | --- | --- | --- | --- |
| **(a)** | ㅡ | 0.5 | 0.5 | 1 | 1 | 1 |
| **(b)** | ㅡ | 2 | ㅡ | ㅡ | ㅡ | ㅡ |
| **(c)** | ㅡ | ㅡ | ㅡ | ㅡ | ㅡ | ㅡ |
| **(d)** | ㅡ | ㅡ | ㅡ | 0.3 | 0.3 | 0.3 |
| **(e)** | ㅡ | 0.5 | 0.2 | 1 | 0.1 | 0.1 |
| **Animal** | **26.5 hr** | **27 hr** | **27.5 hr** | **28 hr** | **28.5 hr** | **29 hr** |
| **(a)** | 1 | 0.5 | 0.5 | 1 | 3 | 32 |
| **(b)** | ㅡ | ㅡ | ㅡ | ㅡ | ㅡ | ㅡ |
| **(c)** | 1.2 | ㅡ | 0.5 | 0.5 | 1.5 | 33 |
| **(d)** | 0.3 | 0.7 | 0.7 | 12.4 | 22.6 | 40 |
| **(e)** | 0.1 | 0.1 | 1.1 | 1 | 24 | 25 |
